# Preclinical efficacy of belinostat in combination with zidovudine in adult T-cell leukemia-lymphoma

**DOI:** 10.1186/1742-4690-12-S1-P73

**Published:** 2015-08-28

**Authors:** Ngoc Toomey, Glen Barber, Juan Carlos Ramos

**Affiliations:** 1 Department of Medicine, Division of Hematology/Oncology, Sylvester Comprehensive Cancer Center, University of Miami Miller School of Medicine, Miami, FL, USA; 2 Department of Cell Biology Sylvester Comprehensive Cancer Center, University of Miami Miller School of Medicine, Miami, FL, USA

## 

Adult T-cell leukemia-lymphoma (ATLL) is an aggressive malignancy that is incurable by conventional drugs. Histone deacetylase (HDAC) inhibitors (HDIs) are broadly active anti-neoplastic agents that can be cautiously exploited in the treatment of ATLL. The HTLV-I provirus is clonally integrated in virtually all ATLL cells. The viral promoter is trans-activated by Tax protein through binding of CREB and p300/CBP at the 5'LTR. Tax binding enhances p300/CBP histone acetyl transferase (HAT) activity resulting in chromatin unwinding and transcription of the viral genome. Such HAT activity is countered by HDACs, and also by HBZ, which unlike Tax is the only viral gene consistently transcribed from the negative 3’ proviral strand end in ATLL. We recently conducted a clinical trial using the old generation HDI valproic acid (VPA) combined with zidovudine (AZT) and interferon alpha (IFNα) during maintenance therapy in patients with ATLL. We hypothesized that the HDI would reactivate HTLV-I thus provoking an immune response against circulating ATLL cells that normally persist after AZT/IFNα therapy. Supporting this notion adding VPA to AZT/IFNα resulted in HTLV-I proviral load reduction, followed by a molecular and prolonged clinical remission in one subject. We recently compared and tested more potent HDIs on primary ATLL cells and low-passage cell lines. In general, HDIs induced dose dependent Tax expression and apoptosis in ATLL cells, and provoked an intracellular innate immune response evidenced by the upregulation of IFN-B and IRF-7. The pan HDI belinostat caused H3 acetylation, blocked HBZ protein expression, and induced Tax protein expression in ATLL cells, while paradoxically inhibiting NF-κB nuclear activity, resulting in concomitant apoptosis. Further, adding AZT to belinostat augmented ATLL cell death. In contrast, IFNα treatment diminished apoptosis induced by belinostat. Based on these preclinical data, we proposed using belinostat in combination with AZT as consolidation therapy for ATLL. A pilot clinical trial is underway at our institution.

